# The effects of electric field and gate bias pulse on the migration and stability of ionized oxygen vacancies in amorphous In–Ga–Zn–O thin film transistors

**DOI:** 10.1088/1468-6996/16/3/034902

**Published:** 2015-05-08

**Authors:** Young Jun Oh, Hyeon-Kyun Noh, Kee Joo Chang

**Affiliations:** 1 Department of Physics, Korea Advanced Institute of Science and Technology, Daejeon 305-701, Korea; 2 CAE Team, Memory Division, Samsung Electronics Co. Ltd, Hwaseong 445-701, Korea

**Keywords:** density functional theory, amorphous In-Ga-Zn-O, oxygen vacancy, oxide thinfilm transistor

## Abstract

Oxygen vacancies have been considered as the origin of threshold voltage instability under negative bias illumination stress in amorphous oxide thin film transistors. Here we report the results of first-principles molecular dynamics simulations for the drift motion of oxygen vacancies. We show that oxygen vacancies, which are initially ionized by trapping photoexcited hole carriers, can easily migrate under an external electric field. Thus, accumulated hole traps near the channel/dielectric interface cause negative shift of the threshold voltage, supporting the oxygen vacancy model. In addition, we find that ionized oxygen vacancies easily recover their neutral defect configurations by capturing electrons when the Fermi level increases. Our results are in good agreement with the experimental observation that applying a positive gate bias pulse of short duration eliminates hole traps and thus leads to the recovery of device stability from persistent photoconductivity.

## 1. Introduction

Recently, Zn-based amorphous oxide semiconductors such as amorphous In–Ga–Zn–O (a-IGZO) have attracted a great deal of attention because these semiconductors are used as channel materials in transparent thin film transistors (TFTs) [1–3]. Despite the high field effect mobility and the low temperature growth with excellent uniformity, the reliability of a-IGZO TFTs still remains an important issue. In particular, a-IGZO TFTs suffer from a high density of charge traps which cause large shifts of the threshold voltage (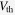) under various stress conditions. Under negative bias illumination stress (NBIS), 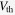 is shifted negatively up to about −18 V; this phenomenon is called the NBIS instability [4–6]. It is known that the NBIS instability persists even after the stress condition is removed, while the device stability is recovered after a long time at room temperature or by thermal annealing [4, 7, 8]. The NBIS instability was thought to be caused by the charge trapping of photoinduced hole carriers [4, 5].

On the basis of first-principles theoretical calculations, it was suggested that O vacancy defects (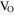) in a-IGZO are responsible for the NBIS instability [9, 10]. In the O vacancy model, it is assumed that 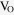 is ionized by light illumination or captures photoinduced holes and then drifts to the a-IGZO/dielectric interface under a negative gate bias. The accumulated hole traps can explain the negative shift of 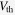. Several experiments have shown that the device stability is improved by high pressure oxygen annealing, oxygen plasma treatment, and ozone treatment [11–14]. Since oxygen and ozone treatments effectively eliminate active 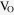 defects, the O vacancy model for the NBIS instability is strongly supported. Moreover, replacing Ga atoms with the group IV elements with high electron negativities, such as Ti, Zr, and Hf, has also yielded improvement of device stability [15–17]. Since oxygen is more strongly bonded to the highly electronegative ions, it costs a higher energy to generate the 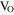 defect. Recently, it was proposed that substituting non-oxygen ions for the O anions increases the valence band edge state and deactivates the deep defect level of 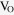, and thus suppresses the NBIS instability [18].

From field-dependent *I*–*V* characteristics of a-IGZO TFTs, it was inferred that ionized 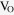 defects easily migrate at room temperature, thus supporting the O vacancy model [19]. However, the details of O vacancy migration during device operation are not well understood. It is also known that the field-induced migration of 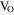 plays a role in oxide-based resistive random-access memory (ReRAM) operation [20–22]. One of the switching mechanisms in ReRAM devices is the formation of a conducting channel under bias, which consists of O vacancy defects [23, 24]. Therefore, it is important to understand microscopically the drift motion of ionized O vacancy defects under an electric field. Once hole carriers are trapped by the 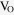 defect, the photoconductivity persists for hours or days, even in the absence of illumination [25], limiting the switching speed of TFTs. In recent experiments, a three-terminal device structure, called a photo-TFT, was used to eliminate the persistent photoconductivity [26]. In this device structure, the position of the Fermi level is controlled by applying a positive gate bias pulse of short duration, allowing for electron capture by ionized O vacancy defects and thus the rapid recovery of device stability.

In this work, we perform first-principles molecular dynamics simulations in order to investigate the drift motion of ionized 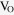 defects under an external electric field in a-IGZO. We also examine the effect of a gate bias pulse on the stability of 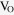 defects within the density functional theory framework. On the basis of the results, we discuss the validity of the O vacancy model for the origin of the NBIS instability and the detailed mechanism for the process of recovery from persistent photoconductivity.

## 2. The calculation method

Our calculations are performed by using the generalized gradient approximation (GGA) for the exchange–correlation potential proposed by Perdew, Burke, and Ernzerhof [27] within the density functional theory (DFT) and the projector augmented wave potentials [28], as implemented in the VASP code [29]. We consider the amorphous phase of InGaO_3_(ZnO)_*m*_ with the composition ratio of In:Ga:Zn:O *=* 1:1:1:4. The defect levels of 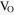 are examined for a supercell containing 84 host atoms. We expand the wavefunctions in plane waves, under periodic boundary conditions, with an energy cutoff of 400 eV and use a 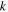-point set generated from the 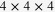 Monkhorst–Pack mesh for Brillouin zone integration [30]. All the ionic coordinates are fully optimized until the residual forces are less than 0.05 eV Å^−1^.

**Figure 1.**
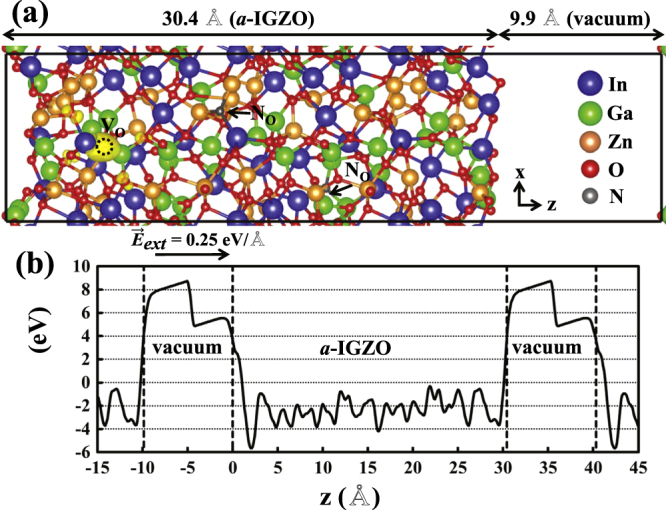
(a) A supercell geometry consisting of a-IGZO layers and a vacuum region containing one 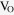 defect and two substitutional N_O_ atoms. (b) The local potential averaged over the *xy* plane plotted along the *z* axis under an external electric field of 0.25 eV Å^−1^.

It is known that, in GGA calculations, the band gaps of semiconductors and insulators are severely underestimated, whereas the positions of metal d bands are overestimated [31]. The on-site Coulomb correlation (*U*) [32], which is described by a Hubbard-like term, is often used to improve the position of metal d bands. With inclusion of the parameters 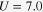, 8.0, and 8.0 eV for the In 4d, Ga 3d, and Zn 3d orbitals, respectively, we reproduce the d-band positions in good agreement with experiments [33, 34]. In the GGA + *U* approach, the band gaps of a-IGZO are still lower than the measured value of about 3.2 eV [35], ranging from 1.44 to 1.78 eV. To improve the band gap size and the formation energies of defects, we also employ the hybrid functional of Heyd, Scuseria, and Ernzerhof (HSE) for the exchange–correlation potential [36, 37]. With the screening parameter of 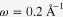 and the mixing fraction of 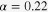, which represents the mixing ratio of the exact short-range Hartree–Fock exchange, we obtain the band gaps of 2.97 ∼ 3.08 eV, close to the measured value. In this case, we take the on-site Coulomb parameters of 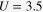, 4.0, and 4.0 eV for the In 4d, Ga 3d, and Zn 3d orbitals, respectively, to reproduce the measured values for the metal d bands. For the drift motion of a charged 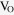 defect, it is difficult to perform molecular dynamics (MD) simulations with the hybrid functional due to the heavy computational demand for the slab geometry (figure 1). Here we point out that the underestimation of the band gap in the GGA + *U* approach would not significantly affect the migration barrier of the charged defect because the defect level remains unoccupied during MD simulations.

We generate three amorphous models through melt-and-quench *ab initio* MD simulations [38], in which a Nosé–Hoover thermostat is used to control the thermal fluctuation in canonical ensembles. Crystalline InGaO_3_(ZnO)_*m*_ is first melted at a temperature of 3000 K for 10 ps and then quenched with the cooling rate of 312.5 K ps^−1^. After repeating the melt-and-quench process, we obtain the amorphous structures and confirm that the 84-atom supercell is sufficient for exhibiting the amorphous character. The details of the structural characteristics for the amorphous phase have been given elsewhere [10].

## 3. Drift motion of the ionized O vacancy under an electric field

The drift motion of the 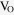 defect is studied by performing *ab initio* MD simulations for a slab geometry which consists of a-IGZO layers of 30.4 Å and a vacuum region of 9.9 Å, as shown in figure 1(a). The bulk region of a-IGZO is constructed by enlarging, to three times the extent, the 84-atom supercell along the direction of the electric field. In the supercell with periodic boundary conditions, an external electric field is allowed by including a dipole correction in the vacuum region [39]. From the plane-averaged local potential (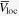) plotted along the *z* axis in figure 1(b), we confirm that a uniform electric field is applied in the bulk region of a-IGZO.

We generate a 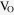 defect by removing one O atom from the a-IGZO slab. In the O vacancy model for the NBIS instability, the defect is in the 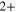 charge state, as a result of capturing hole carriers which are generated by light illumination. The charged 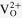 defect drifts toward the a-IGZO/dielectric interface under a negative bias voltage. Thus, we take the 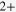 charge state for studying the drift motion of 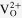. Since periodic boundary conditions are used, the neutral charge state is required for applying an external electric field in the supercell calculations. Otherwise, the electric field is not properly described due to the image charges of 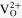. To keep the whole system neutral but the 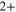 charge state for the 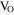 defect, we introduce two N acceptors in the a-IGZO region, which substitute for the two O atoms at 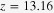 and 19.66 Å. Following this approach, the 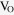 defect maintains the 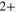 charge state. Note that the N_O_ atoms are only introduced during MD simulations for the drift motion of 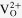 in the slab geometry. We confirm that, during MD simulations, the N 2s and 2p states are fully occupied, so the N_O_ atoms retain the 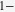 charge state. Moreover, the absence of holes and electrons at the valence and conduction band edges ensures the 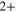 charge state for the 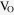 defect. Since the screened potential of N_O_ is weaker than the external potential, we expect the drift motion of 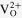 to be not much affected by the N_O_ atoms, but be dominated by the external electric field.

First-principles MD simulations are performed over 19 ps with the time step of 2 fs, with the external electric field set to 0.25 eV Å^−1^. We find that temperature fluctuates around 700 K with the standard deviation of 58 K. During MD simulations, it is difficult to find the trajectory of 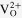 because the defect position is not clearly determined by the coordination numbers of the neighboring metal ions in the amorphous matrix. In previous calculations, the 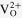 defect was shown to be characterized by the unoccupied defect level lying in the conduction band [9, 10]. We examine the charge distributions of the energy states within 1.0 eV from the conduction band minimum (CBM) in order to identify the position of 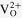. Since the unoccupied defect state is associated with bonding between the metal ions around the vacancy site, the 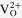 defect is assumed to be positioned in the middle of metal–metal bonds.

**Figure 2.**
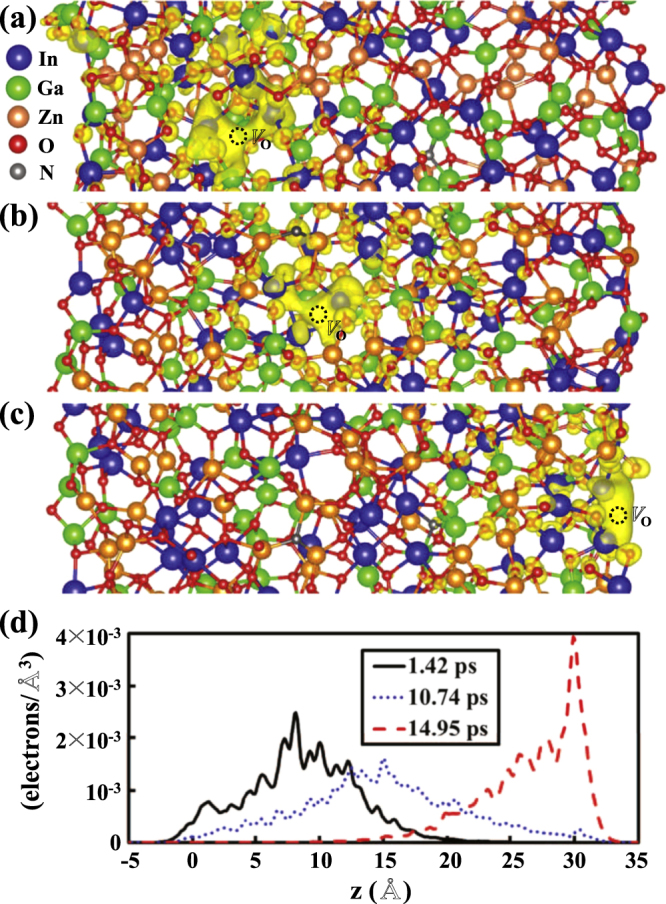
Isosurfaces (yellow) of the charge densities of the defect levels of 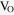 at (a) 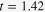 ps, (b) 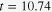 ps, and (c) 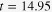 ps. (d) The averaged charge density over the *xy* plane (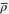) plotted along the *z* axis for the defect level of 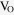 at different times.

To describe more precisely the environment around the O vacancy defect during the drift motion, we use the notion of 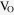
(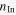 : 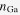 : 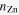), where 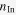, 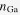, and 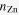 denote the numbers of the neighboring In, Ga, and Zn atoms, respectively. Initially, the 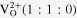 defect, which is surrounded by one In atom and one Ga atom, is formed at 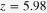 Å. Due to thermal fluctuations, the atoms undergo large relaxations that lead to bond breaking and reconstruction. Thus, at the initial stage of MD simulations, 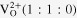 disappears rapidly within 1 ps. Most of the time, we find that the charge densities of the energy states near the CBM are widely distributed over many metal ions because of the hybridization between the defect level and the conduction band states. Although it is generally difficult to trace the trajectory of 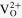, we are able to identify the formation of 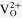 at several different positions, such as 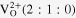 at 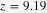 Å and 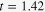 ps, 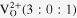 at 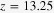 Å and 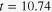 ps, and 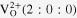 at 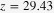 Å and 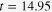 ps. Figure 2 shows the charge distributions of the defect level for identified 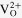 defects. Our results clearly indicate that 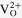 drifts along the direction of the electric field. It is interesting that all of the defects identified are surrounded by more In atoms, with the defect level characterized by the In 5s orbital. This feature is consistent with the previous result that the formation energy of the O vacancy tends to decrease as the number of neighboring In atoms increases [10].

The averaged displacements of the In, Ga, Zn, and O atoms are estimated to be 1.18, 0.89, 1.08, and 1.17 Å, respectively. However, we do not find a linear variation of the mean square displacement with time for each atom. Thus, no significant migration takes place for individual atoms during the period of the MD simulations. On the other hand, we observe that the O vacancy defect migrates rapidly through the slab, although this defect is annihilated and reconstructed at different positions by the collective motion of atoms. The overall movement of the O atoms is correlated with the migration of 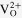. As 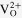 migrates over the distance of 20.24 Å, the total displacement of the O atoms is −9.04 Å, while the individual displacement is at most 2.29 Å. On the basis of the results, it is inferred that the long-distance migration of O vacancy results from the accumulation of short-distance motions of individual atoms.

We estimate the drift velocity (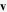) of 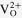 to be 1.29 Å ps^−1^ from the linear fit of the vacancy position versus time at the temperature of *T* = 700 K. The drift motion of an ion under an external electric field (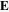) can be described as a simple rigid point-ion hopping process with a field-dependent hopping barrier [40, 41] 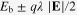, where 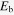 is the energy barrier, *q* is the charge state of the ion, and *λ* is the jump distance. Then, the drift velocity is expressed as

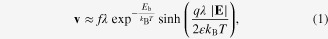
where *f* is the attempt frequency and *∊* is the static dielectric constant. Here we approximate the attempt frequency with the vibrational frequency of the O atoms. We calculate the vibrational spectrum by using the Fourier transform of the velocity autocorrelation function 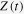, which is defined as

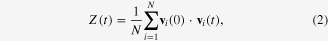
where *N* is the total number of O atoms and 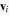 is the velocity of the *i*th O atom. The average vibrational frequency of the O atoms is calculated to be 25.5 THz in a-IGZO. Using 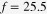 THz, 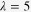 Å adopted for crystalline In_2_O_3_ [42], the measured dielectric constant of 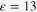 [43], and 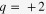 for the charge state of the diffusing species, we obtain the energy barrier of 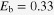 eV. This energy barrier is in good agreement with the measured value of 0.36 eV for the relaxation process of synaptic short-term plasticity in an a-IGZO memristor, which has been attributed to the oxygen migration [44]. However, our resulting value for the amorphous structure is much lower than the previously calculated values for the migration of 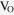 in crystalline In_2_O_3_ (∼0.7 eV) and ZnO (∼1.0 eV) [42, 45].

For a-IGZO TFTs, the results for bias-dependent *I*–*V* characteristics suggested that ionized O vacancy defects migrate at room temperature [19]. Recently, the suppression of NBIS degradation was observed under a large drain voltage, which can be explained by drift and accumulation of ionized O vacancies in the drain region [46]. Along with this experimental evidence, our calculations strongly support the drift motion of ionized O vacancy defects along the direction of the electric field. On the basis of the results, we summarize the mechanism for the NBIS instability as follows. Light illumination induces the ionization of intrinsic 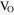 defects by capturing photoexcited holes. Then, ionized 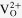 defects tend to drift toward the channel/dielectric interface under a negative bias voltage. Finally, the accumulation of 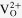 defects near the interface causes a negative shift of 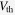.

## 4. Recovery of device stability by gate bias modulation

To mitigate the NBIS instability, it is important to control the formation of the 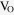 defect in a-IGZO. Previous theoretical calculations [10] showed that adding Ga atoms improves the NBIS instability because the formation energy of 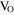 increases with increasing number of Ga atoms around the vacancy site. Experimentally, the NBIS instability has been improved by high pressure oxygen annealing, in which the number of 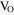 defects is effectively reduced [11–14]. Here we examine the effect of a positive gate bias pulse, which has been used to improve the NBIS instability [26]. The stability of the ionized 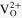 defect will be significantly affected by a positive bias pulse, which increases the Fermi level above the CBM of a-IGZO.

**Figure 3.**
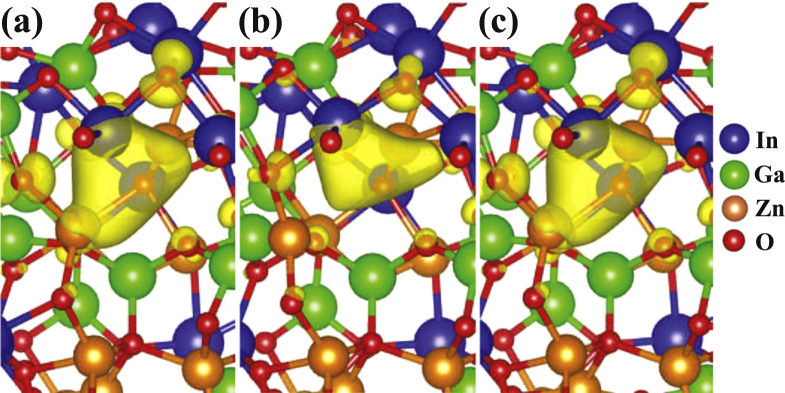
Isosurfaces of the charge densities of the defect levels of (a) the initial neutral 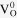 defect, (b) the ionized 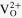 defect, and (c) the final neutral 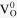 defect, which is obtained by controlling the Fermi level.

We consider 33 different 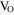 defects which are generated in the three amorphous models [10]. In the neutral state, most of the 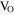 defects (actually, 29 defects) exhibit deep defect levels, with the average level at 0.97 eV above the valence band maximum (VBM). These deep levels result from inward relaxations of the metal ions around the vacancy site, as shown in figure 3(a). When 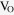 captures two holes, the neighboring metal ions undergo outward relaxations, as shown in figure 3(b), and the unoccupied defect levels move above the CBM. The average value of the unoccupied defect levels is estimated to be 0.58 eV above the CBM. Due to significant outward relaxations, 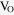 acts as a negative-*U* defect, with the 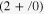 charge transition levels around 0.40 eV above the CBM. On the other hand, we find that four 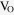 defects are shallow donors, which are attributed to outward relaxations of the neighboring metal ions. Since the defect levels lie above the CBM, these shallow 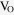 defects are ionized even in the neutral state, donating electrons to the conduction band.

When the Fermi level moves above the defect level, the ionized 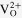 defect will be in principle returned to its neutral configuration by capturing two electrons. Experimentally, the Fermi level can be controlled by applying a positive gate bias pulse for a short duration. To investigate the effect of gate bias control on the stability of 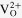, we perform DFT calculations as follows. First, we add *n* electrons to the supercell containing a 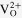 defect and optimize the atomic structure. Second, we remove 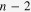 electrons to make the charge state neutral and then optimize the atomic structure again. Finally, we check the position of the defect level in order to determine whether the defect is electrically active or not. If the unoccupied defect level is high, lying well above the CBM, we repeat the same procedure, but increasing the number of added electrons (*n*) until the defect level is occupied, because *n* actually corresponds to the level of the gate voltage pulse. In our calculations, the number of added electrons starts from 2 and then increases up to 10, with an increment of 2 per step. In the final configuration, if 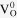 has an occupied defect level deeper than 0.1 eV below the CBM, it is considered a deep defect.

**Figure 4.**
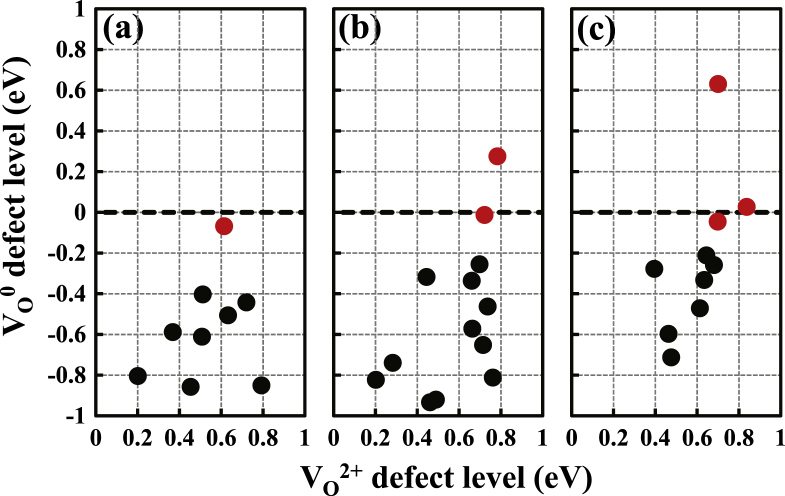
The unoccupied defect levels of 33 ionized 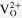 defects in three amorphous structures compared with the occupied defect levels of the neutral 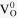 defects within the GGA + *U* calculations, which are obtained by controlling the Fermi level. Dashed lines denote the CBM state which is set to zero. Red circles denote six 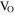 defects which act as shallow donors even after the unoccupied defect level is filled by applying a gate voltage pulse.

Upon controlling the Fermi level as described previously, we notice that most of the 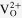 defects are recovered to the deep defect by undergoing inward relaxation, as illustrated in figure 3(c). In figure 4, the unoccupied defect levels of the initial 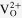 defects are compared with the occupied deep levels of the final 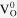 defects within the GGA + *U* calculations, which are recovered by controlling the position of the Fermi level. We find that among the 33 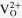 defects considered, 27 have deep defect levels in the neutral state after capturing electrons. The average position of the deep defect levels is estimated to be 1.08 eV above the VBM. The charge density of the defect level is mostly distributed between the metal ions around the vacancy site, as shown in figure 3. Thus, the neighboring metal ions undergo inward relaxations upon electron capture. During the process of recovery of the deep defects, there is no energy barrier to inward relaxations. For the other defects, in which deep defect levels are not formed, we find that they are characterized by the Ga neighbors. When 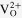 is surrounded by more Ga ions, the unoccupied defect level tends to increase due to the strong Ga–metal bond [10]. In this case, the 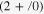 transition level also becomes high, so a high voltage pulse is required to fill the unoccupied defect state. However, in our calculations, we do not observe the recovery of deep defects, probably because of the strong hybridization between the defect level and the conduction band states. Nevertheless, since it is energetically less favorable to generate O vacancies with Ga neighbors, most of the ionized 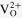 defects will recover their deep defect states under a short bias pulse and eliminate the persistent photoconductivity.

The recovery process is also examined by performing hybrid functional calculations which yield more reliable band gaps and defect formation energies. We select, among the 33 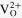 defects, four defects with distinctive environments: 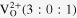, 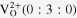, 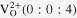, and 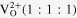. The 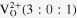, 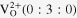, and 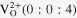 vacancies are characterized by In-, Ga-, and Zn-rich neighbors, respectively, while the 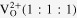 defect is surrounded by one In atom, one Zn atom, and one Ga atom. In the GGA + *U* calculations, three 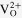 defects recover their neutral states—excluding the 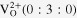 defect with more Ga neighbors, as discussed earlier. In this case, the defect levels of the recovered neutral configurations lie at 0.34–0.82 eV below the CBM. With the hybrid functional, we find that all of the 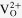 defects considered recover their neutral configurations, with the defect levels at 0.45–1.25 eV below the CBM. Due to the band gap being enlarged by the hybrid functional, the defect levels become deeper and even the 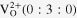 defect recovers its neutral configuration. On the basis of the GGA + *U* and hybrid functional calculations, it is clear that the recovery reaction readily takes place via electron capture for charged 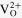 defects.

## 5. Conclusions

In conclusion, we have performed first-principles molecular dynamics simulations for the migration of ionized O vacancy defects in amorphous In–Ga–Zn–O semiconductors and shown that these defects readily diffuse under an electric field, in good agreement with recent experiments. The drift motion of the O vacancy is accompanied with small displacements of the individual O atoms. Our results strongly support the theoretical model in which O vacancy defects are responsible for the NBIS instability observed in a-IGZO TFTs. We have examined the effect of a positive gate bias pulse on the stability of ionized O vacancy defects, and find that most of the defects capture electrons and thereby recover their deep defect states. Thus, the device stability can be restored by applying a short gate bias pulse.
